# Synthesis, Toxicity Assessment, Environmental and Biomedical Applications of MXenes: A Review

**DOI:** 10.3390/nano12111797

**Published:** 2022-05-24

**Authors:** Inna A. Vasyukova, Olga V. Zakharova, Denis V. Kuznetsov, Alexander A. Gusev

**Affiliations:** 1 Technopark “Derzhavinsky”, Derzhavin Tambov State University, 392000 Tambov, Russia; vasyukovaia@gmail.com (I.A.V.); olgazakharova1@mail.ru (O.V.Z.); 2 Department of Functional Nanosystems and High-Temperature Materials, National University of Science and Technology “MISIS”, 119991 Moscow, Russia; dk@misis.ru; 3 Engineering Center, Plekhanov Russian University of Economics, 117997 Moscow, Russia

**Keywords:** 2D materials, MXene, toxicity, biomedicine, environmental science

## Abstract

MXenes are a family of two-dimensional (2D) composite materials based on transition metal carbides, nitrides and carbonitrides that have been attracting attention since 2011. Combination of electrical and mechanical properties with hydrophilicity makes them promising materials for biomedical applications. This review briefly discusses methods for the synthesis of MXenes, their potential applications in medicine, ranging from sensors and antibacterial agents to targeted drug delivery, cancer photo/chemotherapy, tissue engineering, bioimaging, and environmental applications such as sensors and adsorbents. We focus on in vitro and in vivo toxicity and possible mechanisms. We discuss the toxicity analogies of MXenes and other 2D materials such as graphene, mentioning the greater biocompatibility of MXenes. We identify existing barriers that hinder the formation of objective knowledge about the toxicity of MXenes. The most important of these barriers are the differences in the methods of synthesis of MXenes, their composition and structure, including the level of oxidation, the number of layers and flake size; functionalization, test concentrations, duration of exposure, and individual characteristics of biological test objects Finally, we discuss key areas for further research that need to involve new methods of nanotoxicology, including predictive computational methods. Such studies will bring closer the prospect of widespread industrial production and safe use of MXene-based products.

## 1. Introduction

Over almost twenty years two-dimensional (2D) nanomaterials have attracted considerable scientific attention due to their outstanding physical and chemical properties, and their almost unlimited application potential. The list of 2D nanomaterials has been significantly expanded since the first cases of graphene synthesis [1], with materials such as boron nitride, metal dichalcogenides, halogenides, and oxides, and others have been added [2,3,4,5].

MXenes are two-dimensional inorganic compounds consisting of layers of carbides, carbonitrides and nitrides of transition metals a few atoms thick. Discovery in 2011 of the first 2D titanium carbide (Ti_3_C_2_Tx) led to a sharp increase in the number of research works and in the development of the MXenes manufacturing technologies [6]. This fast-growing family of 2D materials, added to the group of two-dimensional materials by Prof. Gogotsi at al. in 2011, now consists of more than 30 compositions derived from MAX phases and represented by layered transition metal carbides, nitrides and carbonitrides [6,7]. MAX phases are a vast group of compounds with the formula Mn + 1AXn, where M is a transition d-metal (Sc, Y, Ti, Zr, Hf, V, Nb, Ta, Cr, Mo or W), A is p-element (Al, S Si etc.) and X is carbon or nitrogen. The major distinctive feature of such phases is their crystalline structure formed by the alternating nanosized layers of d-metal and p-elements [8,9].

## 2. Synthesis

The two main approaches employed for the synthesis of 2D Mxenes are top-down and bottom-up mechanisms. Top-down mechanism correspond to the exfoliation of large crystal quantities into single-layered MXene sheets whereas the bottom-up approach concentrate on the growth of MXenes from atoms/molecules.

**Figure 1:**
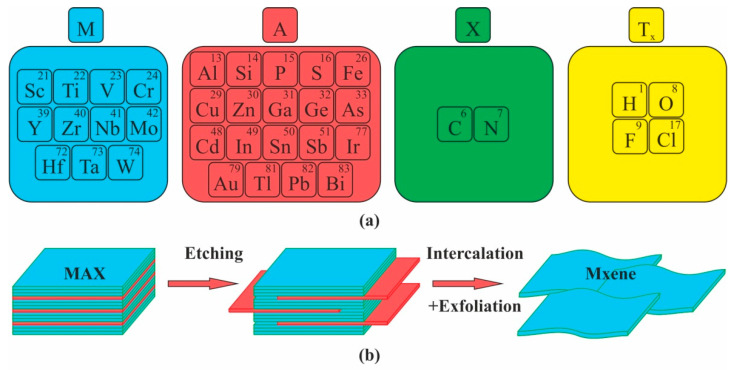
MXenes: (**a**) Constituent elements of MAX and MXenes; (**b**) Top-down synthesis of MXenes from their MAX precursors by selective etching.

To date, much of MXene synthesis has involved the wet application of a MAX phases etching procedure followed by phase exfoliation. MAX phases is a wide class of layered ternary carbides and nitrides exhibiting combination of properties of metallic and ceramic materials obtained by sintering of starting elemental powders [10]. The first and most well-known MXene was Ti_3_C_2_ obtained by dipping Ti_3_AlC_3_ fine powders in presence of hydrofluoric acid at ambient conditions [6]. Thus, all MXenes have the formula Mn + 1XnTx, where M is a transition metal, X is carbon or nitrogen with n = 1, 2 or 3, T are the surface termination groups (represented, as a rule, by O, -F or -OH) and x is the number of the surface functional groups [7,11,12,13] (Figure 1).

Up to date MAX phase etching with hydrofluoric acid (HF) is the most widely used method for fabricating of various types of MXenes [14,15]. However, this technique is not optimal when producing materials intended for use in biomedicine, not only because of HF toxicity for biological objects, but also due to the fact that such MXenes have -F functional groups harmful for some biomedical applications. Li et al. [16] were the first research group to study the possibility of hydrothermal etching of Ti_3_AlC_2_ without fluoride ions, although their process requires an autoclave treatment at 270 °C and the properties of the obtained nanosheets have not yet been studied. Sun et al. suggested another promising method [17] consisting in the MAX phase electrochemical etching with HCl, thus abandoning the use of fluoride, though the process is rather slow and provides only a low yield. Yang et al. [18] recently described yet another promising and highly efficient fluoride-free etching method based on the anodic corrosion of titanium aluminium carbide (Ti_3_AlC_2_) in a binary aqueous electrolyte. The dissolution of aluminium followed by in situ intercalation of ammonium hydroxide results in the extraction of carbide flakes (Ti_3_C_2_Tx, T = O, OH). Thus, the authors not only transformed Ti_3_AlC_2_ into Ti_3_C_2_Tx with a high (over 40%) yield, but also produced the product with the properties comparable with those of the MXenes fabricated by the methods employing HF or HCl/LiF.

In recent years, several alternative MXene synthesis route have been developed, known as bottom-up methods. Among them are the chemical vapor deposition (CVD) [19,20,21,22,23,24], template method [25,26] and plasma-enhanced pulsed laser deposition (PEPLD) [27]. The bottom-up synthesis approach produces higher quality MXenes compared to those manufactured via top-down methods. Additionally, bottom-up methods can grow 2D carbides and nitrides of transition metals with stoichiometry unobtainable through selective etching, including WC [20], TaC and TaN [28] and some heterostructures [24]. It is important to note that bottom-up methods have been unable to produce single-layer structures, and up till now they have yielded only ultrathin films consisting of several layers.

Besides of rapid development of various methods for obtaining of MXenes in scientific laboratories [29,30], the transition from laboratory to industrial production of MXenes by means of top-down synthesis technology has been actively developed in recent years [31,32]. In particular, selective wet etching processes demonstrate good results that makes such MXenes more attractive from the point of practical biomedical and environmental applications.

## 3. Potential Applications in Medicine

**Figure 2:**
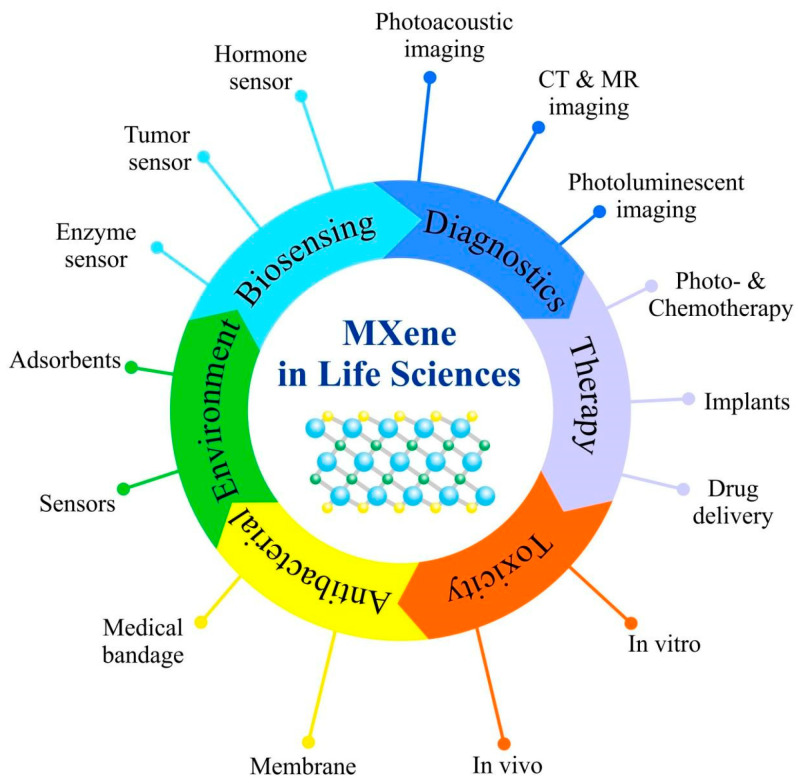
Summary of emerging 2D MXenes used in Life Sciences.

The sphere of MXene applications is constantly growing, and they are now seen as solutions for numerous areas, including optics, manufacturing and energy industries, and biomedicine [33,34,35,36,37,38,39]. Attractiveness of MXenes stems from their outstanding properties, such as high surface-to-volume ratio, excellent electric conductivity, absorption in the near-infrared region, together with the ease with which MXenes surface can be functionalized with various polymers or nanoparticles. All these factors make MXenes suitable nanoplatforms for drug delivery, cancer treatment, bioimaging and biosensor development. Modifications of MXene surface might improve their in vivo effectiveness because of decreased toxicity, improved colloidal stability and prolonged circulation inside the body. MXenes developed for biomedical applications may have structural and dose-dependent antimicrobial activity, and they can be applied in photothermal therapy, addressed drug delivery, photoacoustic and optical imaging, as well as for implant development (Figure 2) [40,41,42].

**Figure 3:**
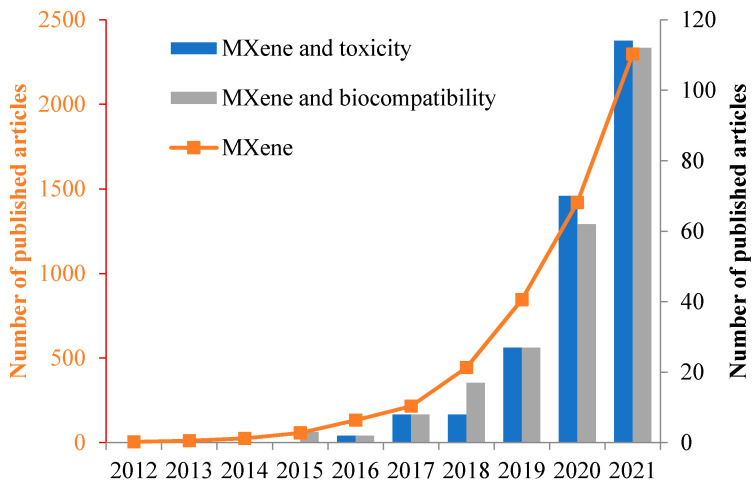
Exponential increase in publications related to MXenes. Data obtained from Scopus using the following search parameters “TITLE-ABS-KEY” (search data 1 March 2022).

Scientific interest in MXenes is steadily increasing (Figure 3). It is clear from the graph that the number of articles on the toxicity/biocompability is growing annually, but their number is less than 5% of the total number of publications on MXenes. This highlights the need for further systematic study of the biological effects of MXenes, given their great potential for biomedical and environmental applications.

### 3.1. Sensors

Currently Ti_2_C and Ti_3_C_2_ MXenes are being used as a basis for developing highly sensitive gas sensors [43,44,45,46,47] and biosensors [48,49,50,51,52,53,54,55,56]. For example, [49,57] studied electrochemical behavior of Ti_3_C_2_ MXenes in mediator-free H_2_O_2_ biosensors. The authors discovered that hemoglobin is adsorbed by the surface functional groups of the nanolayers and becomes immobilized on their inner surfaces, thus making the multi-layered Ti_3_C_2_ structure a promising scaffolding for enzyme immobilization. Rakhi et al. conducted research of a biosensor platform based on Ti_3_C_2_ MXenes for sensitive enzymatic glucose detection [50]. A sensor was fabricated by immobilization of glucose oxidase enzyme on Nafion-solubilized Au/MXene nanocomposite over glassy carbon electrode, and the electrode displayed a linear amperometric response in a very wide glucose concentration range with a relatively high sensitivity as well as excellent stability, reproducibility and repeatability.

**Figure 4:**
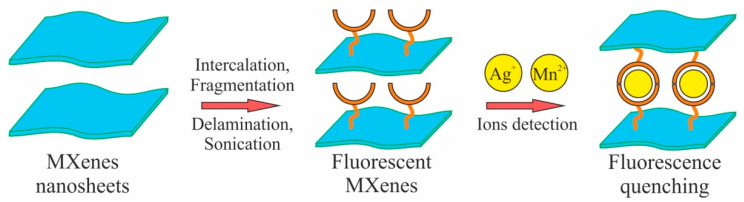
Schematic representation for the preparation of fluorescent MXene nanosheets and their applications for sensitive and selective fluorescence detection of Ag^+^ and Mn^2+^ ions. Based on [59].

Peng at al. [58] developed a simple and highly sensitive sensing platform based on ultrathin two-dimensional MXene Ti_3_C_2_ nanosheets (Ti_3_C_2_ NSs) for selective analysis of Human papillomavirus (HPV-18). Ultrathin Ti_3_C_2_ nanosheets possess high fluorescence quenching ability to dye-labeled single-stranded DNA (ssDNA) and different affinities for ssDNA and double-stranded DNA (dsDNA). This fluorescent sensor for HPV-18 detection shows a low detection limit of 100 pM and a high specificity. Additionally, the developed DNA sensor can be employed to determine PCR amplified HPV-18 from cervical scrapes samples. This work shows that ultrathin Ti_3_C_2_ nanosheets can be potential candidates for construction of high-performance fluorescence DNA biosensors. A similar principle of fluorescence quenching has been employed for developing another sensor for selective detection of Ag^+^ and Mn^2+^ ions (Figure 4) by means of fluorescence quenching of nanosized Ti_3_C_2_ MXene [59].

Monolayer MXene Ti_3_C_2_ shows great ability to sense Ag^+^ and Mn^2+^ ions due to its good hydrophilicity and the presence of functional groups on its surface. The synthesized Ti_3_C_2_ nanosheets display highest emission fluorescence peak at 461 nm upon the excitation wavelength of 384 nm. The quenching of the fluorescence emission peak of Ti_3_C_2_ was observed only upon the addition of Ag^+^ and Mn^2+^ ions, exhibiting good linear response between I0/I and concentration in the range of 0.1–40 μM and 0.5–60 μM for Ag^+^ and Mn^2+^ ions. The authors consider the proposed method useful for detecting Ag^+^ and Mn^2+^ ions in food and real water samples.

A nanohybrid of Ti_3_C_2_Tx MXene and phosphomolybdic acid (PMo12) embedded with polypyrrole (PPy@Ti_3_C_2_Tx/PMo_12_) was presented by Zhou at al. [60] as an aptamer biosensor for osteopontin (OPN) detection. The obtained PPy@Ti_3_C_2_Tx/PMo_12_ hybrid not only displayed rich-chemical functionality, relatively high crystallinity degree, and homogeneous surface morphology but also showed desirable electrochemical activity. These features provided the hybrid with good stability, excellent biocompatibility, and strong binding force toward OPN aptamer strands. A PPy@Ti_3_C_2_Tx/PMo_12_-based aptasensor exhibited an extremely low detection limit of 0.98 μg/L as well as high selectivity and stability, good reproducibility, acceptable regenerability, and applicability in human serum samples. These properties make the hybrid a sensitive and reliable tool for OPN detection in clinical diagnostics.

A biosensor based on Pt nanoparticles-modified Ti_3_C_2_Tx could detect small redox molecules such as ascorbic acid, dopamine, uric acid and acetaminophen with selectivity down to nM level [61].

Ti_3_C_2_Tx MXenes with a few layers also display excellent results when used as a novel highly-sensitive surface plasmon resonance biosensor. Wu at al. [62] demonstrated that coating of the metals (Au, Ag, Cu, Al) with a thin Ti_3_C_2_Tx MXene film enhances the SPR-biosensor sensitivity at λ = 633 nm by 16.8–46.3%, depending on the metal and on the number of MXene layers.

**Figure 5:**
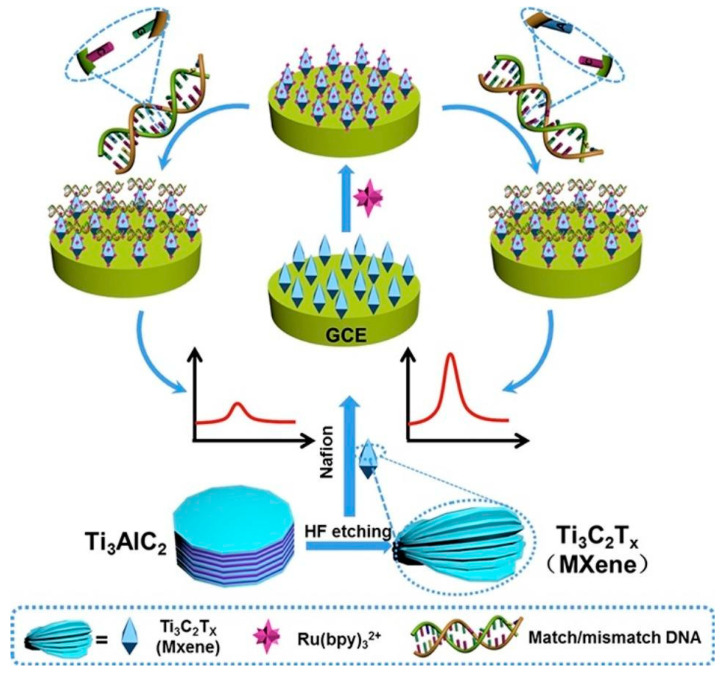
Schematic diagram of solid-state sensor preparation on glass carbon electrode (GCE) based on Ti_3_C_2_Tx MXene obtained from HF-etching Ti_3_AlC_2_ and single-nucleotide mismatch discrimination by the prepared sensor. Reprinted with permission from Ref. [63]. 2022, Elsevier.

In addition, Ti_3_C_2_Tx can be used as an electroluminescent (ECL) sensor for nucleotide mismatch discrimination in human urine samples [63]. A solid-state ECL sensor was prepared by depositing Ti_3_C_2_ on a glass carbon electrode (GCE). The Ti_3_C_2_Tx coating enhanced Ru(bpy)32+ adsorption on the electrode surface (Figure 5).

The sensor was examined using tripropylamine (TPA) as a representative ECL coreactant. The sensor was shown to be applicable for detecting a single-nucleotide mismatch in the p53 gene, which proves MXenes highly useful for cancer diagnostics and for other biomedical applications.

**Figure 6:**
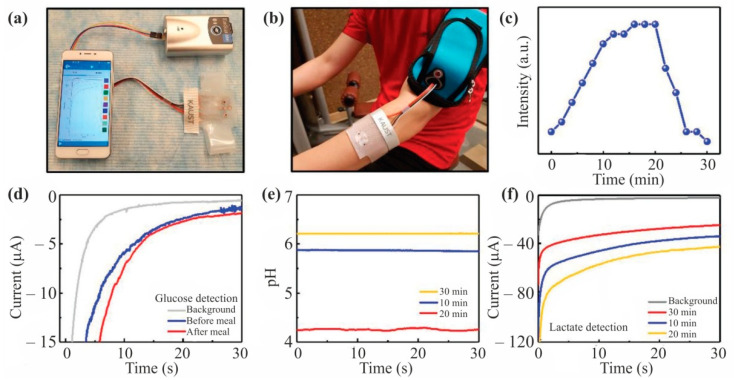
Real-time on-body evaluation of the MXene-based sensor patch indicating the pH levels, lactate, and glucose concentrations. (**a**) Schematic illustration of the oxygen-rich enzyme electrode. (**b**) The wearable sweat-monitoring patch is connected to a portable electrochemical analyzer on the skin. (**c**) Cycling resistance profile for on-body tests. (**d**) Measured chronoamperometric responses of glucose sensors and pH changes before and after meals with three different glucose sensors. (**e**) Measured pH level of pH sensor at different times during the exercise. (**f**) Measured chronoamperometric responses of the lactate sensor at different times during the exercise. Reprinted with permission from Ref. [64]. 2022, John Wiley and Sons.

Wearable perspiration analyzer can become the next step in non-invasive monitoring of health biomarkers. Lei et al. [64] developed a stretchable, wearable, and modular multifunctional biosensor incorporating a novel MXene/Prussian blue (Ti_3_C_2_Tx/PB) composite designed for sensitive detection of biomarkers (e.g., glucose and lactate) in sweat (Figure 6). A three-phase solid-liquid-air interface guarantees superior sensor performance and stability in in vitro experiments.

Cheng at al. [65] employed an abrasive paper stencil printing process to produce a highly sensitive MXene-based piezoresistive sensor with bioinspired microporous microspinous structures. The fabricated sensor showed high sensitivity (151.4 kPa^−1^), relatively short response time (<130 ms), subtle pressure detection limit of 4.4 Pa, and excellent cycle stability over 10,000 cycles. In practice, the sensor showed great performance in monitoring human physiological signals, detecting quantitatively pressure distributions, and remote monitoring of intelligent robot motion in real time.

Liu at al. [66] reported development of a MXene-based microfluidic biosensor. A Ti_3_C_2_Tx based screen-printed electrode incorporated with a dialysis microfluidic chip was constructed for a direct and continuous multicomponent analysis of whole blood. The fabricated sensor can be applied for continuous assay of urea, uric acid, and creatinine levels in human blood.

Determination of hepatotoxic drugs is critical for both clinical diagnosis and quantity control of their pharmaceutical formulations. Zhang at al. [67] described the developed a simple but sensitive sensor based on an MXene modified screen-printed electrode (MXene/SPE) for detection of acetaminophen (ACOP) and isoniazid (INZ), which are two commonly used drugs that might, in certain circumstances, induce liver damage. MXene modified SPE showed excellent electrocatalytic activity toward the oxidation of ACOP and INZ compared with bare SPE in 0.1 M H_2_SO_4_, and the separated oxidation peak potentials ensured simultaneous detection of the targets within wide linear ranges from 0.25 to 2000 μM for ACOP and from 0.1–4.6 mM for INZ. The detection limits of ACOP and INZ were 0.048 μM and 0.064 mM, respectively.

The comparative efficiency of biomedical sensors based on MXenes is presented in Table 1.

### 3.2. Targeted Drug Delivery

Targeted drug delivery is the delivery to a target site without affecting other tissues. In targeted drug delivery, bioavailability is one of the important issues. One of the factors increasing the bioavailability of drugs is their hydrophilicity. Therefore, hydrophilic MXenes are good candidates for a targeted delivery platform. Another important advantage of MXenes is the ability of their surface to be functionalized with therapeutic molecules.

An MXene-based platform for targeted drug delivery can become a useful addition to the arsenal of cancer treatment methods [74,75,76]. Liu et al. [77] described a method for layer-by-layer Ti_3_C_2_ surface modification with doxorubicin and hyaluronic acid, that creates an effective platform for selective chemo/photothermal cancer therapy.

Xing et al. [78] were the first to synthesize composite hydrogels based on cellulose and Ti_3_C_2_ MXene for loading with anticancer drugs and their delivery to malignant cells. This nanoplatform provides combined chemo/photothermal cancer therapy. Properties such as large pores and high water content (98%) mean that the material is characterized by high drug-loading capability (84%). Good biocompatibility and three-dimensional networks of the hydrogel promote controlled sustained release of doxorubicin hydrochloride, thus reducing the drug toxicity. The authors reported that the cellulose/MXene composite hydrogels possess excellent infrared absorption characteristics, especially well displayed under illumination with an 808 nm wavelength. The response to illumination manifests itself as a continuous dynamic process in water and promotes drug release due to expansion of the pores. After laser irradiation for 5 min the hydrogel with 235.2 ppm MXene concentration led to 100% non-relapsive death of the tumor cells with the cell biodegradation within two weeks.

Ti_3_C_2_ MXenes and composite materials based on them have a high drug-loading capacity [79,80]. In addition, according to Han et al. [80] Ti_3_C_2_ MXenes not only possess drug-loading capability as high as 211.8%, but also exhibit both pH-responsive and near infrared laser-triggered on-demand drug release. The authors explored the Ti_3_C_2_ MXenes ability for efficient tumor eradication by synergistic photothermal ablation and chemotherapy, which was systematically demonstrated both in vitro and in vivo. These Ti_3_C_2_ MXenes have also been demonstrated as desirable contrast agents for photoacoustic imaging, showing the potential for diagnostic-imaging guidance and monitoring during therapy. The high in vivo histocompatibility of Ti_3_C_2_ and its easy excretion out of the body have been evaluated and demonstrated, showing high biosafety for further clinical translation.

The advantage of MXenes for targeted drug delivery is their hydrophilicity, which increases the bioavailability of drugs for body tissues [81], high drug loading capacity [82], as well as facile encapsulation [83]. This makes MXenes one of the most promising new materials for biomedicine, including various applications for combating COVID-19 [84].

### 3.3. Photo/Chemotherapy of Cancer

The development of therapies that are selective for tumor tissues is one of the most important goals of anticancer research. Within this framework, photo- and chemotherapy can be considered a very promising approach. These approaches require fluorosensitizers and chemotherapeutic agents that are bioavailable and nontoxic to the surrounding tissues. MXenes provide a promising basis for such pharmaceuticals.

MXenes can be successfully used as novel highly efficient and selective agents for photothermal cancer therapy [76,77,85,86].

Ti_2_C MXenes superficially modified with PEG showed a good photothermal conversion efficacy thus triggering cancerous cells’ ablation with a satisfactory selectivity towards non-malignant cells during in vitro experiments. The observed effects might be due to MXene-induced reactive oxygen intermediates production generated by the photothermal effect. The applied doses of Ti_2_C_PEG in the presented work were considerably lower compared to other MXene-based photothermal agents [85]. Lin et al. [87] modified Ti_3_C_2_Tx MXene with soybean phospholipid (SP) and with poly(lactic-co-glycolic acid) (PLGA) in order to reveal the effects of the hybrid as a photothermal agent for cancer treatment. Both in vitro and in vivo experiments proved a high potential of modified Ti_3_C_2_ MXenes as a novel photothermal agent for cancer therapy, providing excellent relapse-free tumor ablation both in the case of Ti_3_C_2_/SP intravenous administration at 20 μg/kg and in the case of localized intratumoral implantation of PLGA/Ti_3_C_2_ at 2 μg/kg. It is important to note that the characteristics of the PLGA/Ti_3_C_2_-SP phase transition not only eradicate the tumor but also ensure no escape of the implanted agents into the bloodstream, thus making the studied material safe for in vivo applications.

Despite a large number of papers on photonic tumor hyperthermia, current photothermal-conversion nanoagents still suffer from critical issues preventing further clinical translation, including low biodegradability. In their work Feng et al. [88] report the construction of novel 2D molybdenum carbide (Mo_2_C) MXenes for photothermal tumor hyperthermia. Surface treatment of Mo_2_C-PVA nanoflakes with polyvinyl alcohol (PVA) confers high biocompatibility and fast degradability. One should note that Mo_2_C-PVA MXene possesses intense near-infrared (NIR) absorption, covering the near-infrared region (NIR I and II), and a desirable photothermal-conversion efficiency (24.5% for NIR I and 43.3% for NIR II). This study not only broadens the nanomedical applications of MXene, but also provides the paradigm of an inorganic multifunctional biomedical nanoplatform with desirable biodegradability and high therapeutic performance.

Biocompatible Ta_4_C_3_ MXenes exhibit unique functionalities for photothermal conversion and for in vitro/in vivo photothermal ablation of tumors. Lin et al. [89] developed a multifunctional nanosystem based on 2D tantalum carbide (Ta_4_C_3_ MXenes) modified with soybean phospholipid (SP) for dual mode photoacoustic/KT imaging and highly effective in vivo photothermal tumor ablation in murine xenograft models. Two-dimensional ultrathin Ta_4_C_3_-SP nanosheets with lateral sizes ≈100 nm displayed outstanding photothermal characteristics in the near-infrared region with the attenuation coefficient 4.06 Lg^−1^cm^−1^ at 808 nm, superior photothermal-conversion performance (44.7%), as well as photothermal stability. It is significant that Ta_4_C_3_-SP nanosheets did not display toxic effects during in vitro or in vivo experiments.

The 3D scaffolds integrating 2D Ti_3_C_2_ MXene into 3D-printed bioactive glass structures [86] seem highly promising for the treatment of bone tumors, as they induce photothermal bone-tumor ablation and improve bone-tissue regeneration.

Traditionally, ceramic-based materials, produced by high-temperature solid-phase reaction and sintering, are preferred as bone scaffolds in hard-tissue engineering because of their tunable biocompatibility and excellent mechanical properties. However, their possible cancer phototherapeutic applications in the near-infrared light (NIR-I and NIR-II) have rarely been considered. The study of a novel kind of MXene, namely 2D niobium carbide (Nb_2_C), has become among the first research works in this area [90]. The authors demonstrated high effectiveness of the material both in NIR-I and NIR-II biowindows. The ultrathin Nb2C nanosheets exhibited extraordinarily high photothermal conversion efficiency (36.4% at NIR-I and 45.65% at NIR-II), as well as high photothermal stability during in vivo photothermal ablation of murine xenograft tumors. The Nb_2_C nanosheets intrinsically feature unique enzyme-responsive biodegradability to human myeloperoxidase, low phototoxicity, and high biocompatibility.

MXenes can become a solution to the therapy of tumors insensitive to traditional chemotherapy, for example, as in hepatocellular carcinoma (HCC), which is one of the most common and deadly gastrointestinal malignancies. Li et al. reported development of a novel 2D MXene-based composite nanoplatform for highly efficient and synergistic chemotherapy and photothermal hyperthermia against HCC. A surface-nanopore engineering strategy was developed for the MXenes’ surface functionalization, which achieved the uniform coating of a thin mesoporous-silica layer onto the surface of 2D Ti_3_C_2_ MXene (Ti_3_C_2_@mMSNs). Both in vitro and in vivo experiments demonstrated high active-targeting capability, synergistic chemotherapy (contributed by the mesoporous shell) and photothermal hyperthermia (contributed by the Ti_3_C_2_ MXene core), resulting in complete eradication of the tumor without obvious reoccurrence [91].

Titanium carbide (Ti_3_C_2_) MXene quantum dots (MQDs) possess intrinsic immunomodulatory properties and selectively reduce activation of human CD4+ IFN-γ+ T-lymphocytes by ≈20%, simultaneously promoting expansion of immunosuppressive CD4+ CD25+ FoxP3+ regulatory T-cells by 3% in a stimulated lymphocyte population [92]. Furthermore, MQDs are biocompatible with bone marrow-derived mesenchymal stem cells and induced pluripotent stem cell-derived fibroblasts.

### 3.4. Tissue Engineering

Tissue engineering includes techniques that enhance or replace biological tissues using a combination of cells, engineered materials, and appropriate biochemical and physicochemical factors. Tissue engineered materials must be biocompatible and have a set of specific mechanical properties. Therefore, fabrication of tissue engineered matrices is another application that can successfully utilize such MXene properties such as mechanical strength, biocompatibility and excellent electroconductivity. Zhang et al. [93] studied osteoinductivity and guided bone regeneration ability of multilayered Ti_3_C_2_Tx MXene films in vitro and in vivo. The research work showed that MXene films are highly cytocompatible and enhance osteogenic differentiation in vitro. When implanted into subcutaneous sites and calvarial defect sites in rats, MXene films showed good biocompatibility, osteoinductivity and bone regeneration activity in vivo. In particular, the authors observed increased activity of macrophages attached to the MXene films which might indicate initiation of MXenes biodestruction in the body.

In the work of Huang et al. [94] composite MXene-containing nanofibers were fabricated by electrospinning and doping, and displayed excellent hydrophilicity because of a large number of introduced functional hydrophilic groups. The conditions proved to provide a good microenvironment for bone marrow-derived mesenchymal stem cells (BMSC) growth. The experiment results demonstrated that the obtained MXene composite nanofibers had good biocompatibility and greatly improved cellular activity by enhancing mesenchymal stem cells differentiation to osteoblasts.

Pan et al. [86] evaluated the effect of a 3D matrix consisting of Ti_3_C_2_ MXene and bioglass on osteoblast cell osteogenic potential. The results showed that these Ti_3_C_2_ MXene-integrated composite scaffolds efficiently accelerated growth of newborn bone tissue while providing it with a good adhesion medium. The authors noted excellent development of the cell filopodia fiber, enhanced number of calcium nodules and moderate induction of cell proliferation. Further study conducted on Sprague–Dawley rats showed that MXene integration into the 3D scaffolding enhanced the osteogenesis rate in the damaged bone area by 30% compared to MXene-free scaffolding.

Thus, these results prove that MXenes can become an excellent material for tissue engineering and controlled bone tissue regeneration.

### 3.5. Bioimaging

Bioimaging is a non-invasive biological activity visualization process that does not interfere with various life processes and helps to explore the three-dimensional structure of samples. Quantum dots are essential components of bioimaging systems. The biocompatibility of quantum dots makes it possible to use them in a biological environment. MXenes are capable of becoming the basis for the fabrication of such quantum dots.

Exceptional properties of Ti_3_C_2_ MXene-based quantum dots have shown great promise for their employment as fluorescent sensors in bioimaging, optical sensing, and photoelectric conversion. [95].

Ti_3_C_2_Tx were successfully used as biocompatible multicolor sensors for photoluminescent detection of RAW264.7 cell lines, which may greatly extend the applications of MXene-based materials in optical sensing. MXene quantum dots (MQD) showed excitation-dependent photoluminescence spectra with quantum yields of up to ≈10% due to strong quantum confinement [96].

Lu et al. [97] demonstrated a facile, high-output method for preparing bright white emitting Ti_3_C_2_ MQDs. The resulting product was two layers thick with a lateral dimension of 13.1 nm. Importantly, the Ti_3_C_2_ MQDs presented strong two-photon white fluorescence. Their fluorescence under high pressure was also investigated and the team found that the white emission was very stable and that pressure application made it possible to change emission from cool white to warm white.

**Figure 7:**
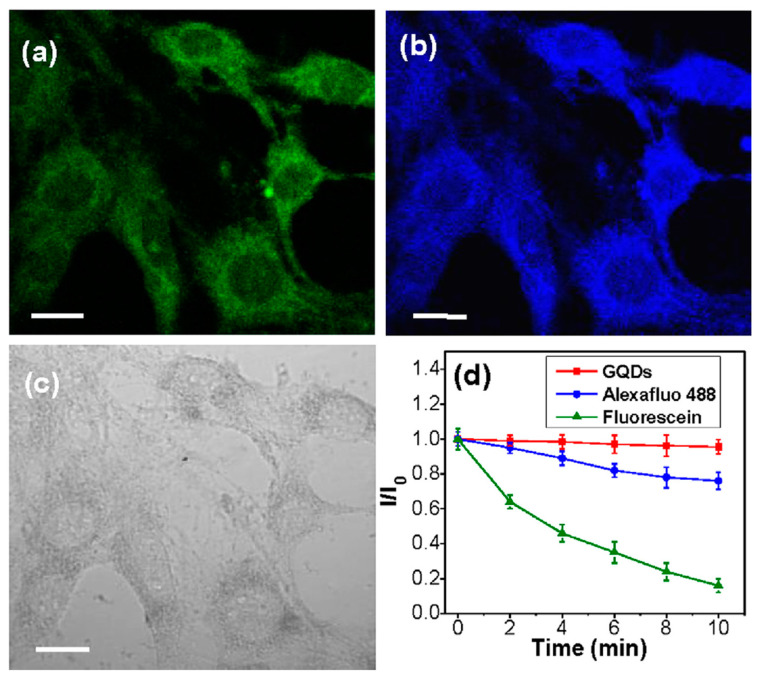
CLSM images of MCF-7 cells after incubation with MXene-based GQDs for 2 h by excitation at 488 nm (**a**), 364 nm (**b**), and under bright field (**c**). The scale bar is 20 mm. (**d**) Time-dependent fluorescence intensity ratio (*I/I_0_*) of GQDs, Alexa fluo 488, and fluorescein. *I_0_* and *I* are the emission intensities of GQDs, Alexa fluo 488, and fluorescein without and with laser illumination for diverse time, respectively. Reprinted with permission from Ref. [98]. 2022, Elsevier.

Zhou et al. [98] reported an unprecedented method for the synthesis of amphiphilic carbide-derived graphene quantum dots (GQDs) from layered Ti_3_C_2_Tx MXene using solvothermal treatment of Ti_3_C_2_Tx MXene in dimethylformamide (DMF). The research results indicate that DMF can simultaneously act as reaction media and nitrogen-doping agent for the formation of highly fluorescent carbide-derived GQDs. The resulting GQDs, with uniform size distribution, exhibit excellent dispersibility in both hydrophilic and hydrophobic solvents. With their superior properties of bright and tunable photoluminescence, low cytotoxicity, good photostability and chemical inertness, the carbide-derived GQDs are promising for applications in fluorescent ink, light-emitting composites and cellular imaging (Figure 7).

Low quantum yield in the UV spectrum, potential instability and nonspecific adsorption reducing the efficiency of MQDs use in the biological environment are the issues that hamper MXenes application for bioimaging. The electron structure-related mechanisms of MXenes remain unclear [99]. Finally, as with other promising nanomaterials in biomedicine, the problems associated with acute and long-term toxicity have not yet been resolved; this point will be discussed in more detail in the following sections.

### 3.6. Antibacterial Agents

For antibacterial agents selective toxicity to bacteria, prevention of antibiotic resistance, and non-toxicity to humans are important. MXenes have a great future in such applications [100,101,102,103]. In particular, 2D materials, including MXenes, are considered to become promising novel antibacterial agents. Their power for disinfection is derived from their unique physicochemical properties and good biocompatibility [104,105]. For example, Ti_3_C_2_/chitosan composite nanofibers are promising candidate materials for creating biodegradable wound dressings [106] effective against wound inoculation with both Gram-positive and Gram-negative bacteria.

Notwithstanding considerable achievements of modern medicine, there is still high annual mortality from various infections, such as dysentery and pneumonia. Excessive and uncontrollable use of broad-spectrum antibiotics has resulted in bacteria gradually developing resistance to antimicrobial agents [107]. Thus, development of new bactericidal compositions is a paramount task for the modern researchers. New 2D materials with unique physicochemical properties that have emerged in the recent decade are paving the road to create highly efficient antibacterial agents [108]. Enhanced membrane permeability, membrane disruption, metabolic activity suppression, DNA destruction, and cell membrane stress caused by its mechanical damage with sharp nanosheets edges, are considered the major mechanisms of 2D nanomaterials antibacterial activity [109,110]. Certain chemical manipulations and functionalizations render MXenes convenient vehicles for various antibacterial functional groups, thus making MXenes a promising class of materials for bacterial and fungal growth inhibition. However, current information on their antimicrobial properties is extremely scarce.

Research conducted by Jastrzebska et al. [111,112] has demonstrated that Ti_2_C displays no toxic effect against Gram-positive bacteria *Bacillus* sp., *Staphylococcus aureus* and *Sarcina*. SEM-examination of the areas of prevailing bacterial absorption revealed insignificant degree of apoptosis only in *Bacillus* sp., especially when the cells were situated between the Ti_2_C layers. Besides, bacterial cells absorption on Ti_2_C nanosheets changed their zeta-potential compared to the native bacterial cells.

The research conducted by Rasool et al. [109,113] shows that Ti_3_C_2_ MXenes display antibacterial properties (up to 99%) against bacterial strains of Gram-positive *Escherichia coli* and Gram-negative *Bacillus subtilis*. Similar results were obtained in the study of the antibacterial properties (up to 100%) of double transition-metal TiVCT_X_ MXene. The authors suggest mechanical damage to the cell membrane as the main mechanism of action. [114].

Based on the data obtained via the colony count method, the descending order of antibacterial activity against both bacterial strains is as follows: single layer Ti_3_C_2_Tx ≫ multilayer Ti_3_C_2_T_x_ > Ti_3_AlC_2_, displaying a clear correlation between the MXenes thickness and their antibacterial activity. Higher dosage of Ti_3_C_2_Tx resulted in sharp decrease in the number of *E. coli* and *B. subtilis* colonies. The authors also report that the antibacterial effect of the studied MXene is even higher than that of graphene oxide due to higher MXene electroconductivity. The authors assume mechanical damage to the cell walls as the primary destructive mechanism. The results of these works suggest dependence of MXene toxicity against bacterial cells on their stoichiometry (i.e., Ti_2_C or Ti_3_C_2_).

In the work of Mayerberger et al. [106] cytotoxicity of a chitosan/Ti_3_C_2_Tx composite was tested against *E. coli* и *S. aureus* strains. The authors report a sharp decrease in the number of the colony forming units by 95% and 62%, respectively; the effect was observed after a 4h treatment with the composite loaded with 0.75wt% Ti_3_C_2_T_x_.

Scaffolds based on Ti_3_C_2_T_x_ MXene@polydopamine demonstrated excellent antibacterial activity against *E. coli*, *S. aureus* and methicillin-resistant *S. aureus* (antibacterial efficiency was 99.03%) [115].

Pandey et al. [116] fabricated Ti_3_C_2_Tx MXene-based membranes loaded with variable amounts of Ag (AgNP) nanoparticles for ultrafast water purification. It is interesting to note that AgNPs were sandwiched between the MXene layers and formed 1–4 nm slits. Both pristine and functionalized with 21% of AgMPs membranes with 470 nm thickness and 2.1 nm pores were investigated for their antibacterial properties against *E. coli*. A hydrophilic polyvinylidene difluoride (PVDF)-based membrane was used for the control. The 21% Ag + MXene composite membrane demonstrated more than 99% *E. coli* growth inhibition, while the pristine Ti_3_C_2_Tx MXene membrane exhibited only ∼60% bacterial growth inhibition compared to the control. Such a pronounced increase in antibacterial activity may be attributed to AgNP influence.

## 4. Environmental Applications

The rapidly developing area of application of MXenes in environmental protection has attracted a lot of attention from researchers. Several recent review articles have focused on this topic [55,117].

MXene-based sensors are successfully used for detection of heavy metal ions, pesticides, and phenols, among other compounds. [118,119,120].

Rasheed et al. [121] suggested a versatile and ultra-sensitive sensor platform based on lamellar Ti_3_C_2_Tx (MXene)-modified glassy carbon electrode. The developed sensor displayed a linear response for the BrO_3_ − concentration from 50 nM to 5 μM with a detection limit of 41 nM.

Fartas et al. [122] developed an electrochemical biosensor based on immobilization of tyrosinase onto graphene-coated gold nanoparticle/chitosan (Gr-Au-Chit/Tyr) nanocomposite-modified screen-printed carbon electrode (SPCE) for the detection of phenolic compounds. The biosensor showed linearity towards phenol in the concentration range from 0.05 to 15 μM with sensitivity of 0.624 μA/μM and a limit of detection of 0.016 μM. The proposed sensor also provided good reproducibility, selectivity and stability for at least one month. The biosensor was compared with a high-performance liquid chromatography (HPLC) method for the detection of phenol in water samples and the result was in good agreement and comparable in efficiency.

Zhu et al. [123] suggested a novel approach to heavy metal detection using MXenes. Two-dimensional accordion-like alk-Ti_3_C_2_, prepared by acid etching and alkaline intercalation treatment, was demonstrated as a new platform for the simultaneous electrochemical detection of multiple heavy metal ions using square wave anodic stripping voltammetry. The method had high sensitivity and good linear correlations, with a detection limit of 0.098, 0.041, 0.032 and 0.130 μM for Cd(II), Pb(II), Cu(II) and Hg(II), respectively. Furthermore, mutual interference among the four target metal ions was explored, and the preferential deposition of Pb(II) in the presence of other three metal ions together with an enhanced Hg(II) sensitivity in the presence of Cd(II) were discovered.

In the work of Song et al. [124], a novel electrochemical sensing platform based on MnO_2_/Mn_3_O_4_ and Ti_3_C_2_ MXene/Au nanoparticles composites were fabricated for ultra-sensitive determination of organophosphorus pesticides. The 3D MnO_2_/Mn_3_O_4_ hierarchical microcuboids derived from Mn-metal-organic frameworks composed of vertically aligned, highly ordered nanosheets, and further combined with MXene/Au nanoparticles, yielded synergistic signal amplification effect while possessing outstanding electrochemical characteristics, large specific surface area, and good environmental biocompatibility. Under optimum conditions, the reported sensing platform AChE-Chit/MXene/Au NPs/MnO_2_/Mn_3_O_4_/GCE can be utilized to detect methamidophos in a broad concentration range (10^−12^–10^−6^ M), together with a good linearity (R = 0.995). Additionally, the biosensor possesses a low limit of detection (1.34 × 10^−13^ M), which far exceeds the maximum residue limits (MRLs) for methamidophos (0.01 mg/kg) established by the European Union.

**Figure 8:**
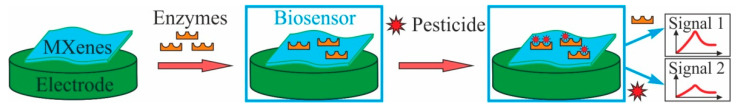
Schematic of MXene-based biosensor for pesticides detection.

In another research paper Zhou et al. [125] describe an AChE (acetylcholinesterase) biosensor based on transition metal carbide nanosheets and chitosan developed for organophosphate pesticides detection. The AChE/CS-Ti_3_C_2_Tx/GCE biosensor demonstrated good characteristics in detection of malathion with a linearity in the range of 1 × 10^−14^–1 × 10^−8^ M, while the detection limit was found to be 0.3 × 10^−14^ M. A schematic diagram of a MXene-based biosensor for pesticide detection is shown in the Figure 8.

Ti_3_C_2_Tx nanocomposites synthesized via a reduction process using the transition metal carbides (MXenes) were used as nanocarriers for electrochemical detection of organophosphate pesticides [126]. The developed biosensor, which combined the unique electrocatalytic properties and synergistic effects between Ti_3_C_2_Tx nanosheets and Ag nanoparticles, not only facilitated electron transfer but also enlarged the available surface area for pesticide detection. Under optimum conditions, the AChE biosensor showed favorable affinity for acetylthiocholine chloride and the corresponding apparent Michaelis-Menten constant (K_mapp_) value was 257.67 μM. The AChE biosensor detected malathion in the linear range from 10^−14^ to 10^−8^ M. In addition, the developed AChE biosensor exhibited satisfactory selectivity, acceptable reproducibility and good stability, that makes it applicable for malathion detection in real samples.

In general, the problems that now hinder the practical implementation of MXenes based sensor include the lack of knowledge of promising samples based not only on traditional titanium but on Mo, V and Nb, as well as the need for ease of functionalization, increasing the efficiency, and stability of MXenes [127].

The comparative efficiency of the discussed sensors is presented in Table 2.

MXenes can be used as highly efficient adsorbents [132,133,134]. Ti_3_C_2_Tx MXene serves as a good example with its high selectivity of adsorption of such pollutants as lead, copper, chromium [135,136,137,138], barium [139], mercury [140], and pharmaceutical compounds [141,142]. Notably, the adsorption efficiency of Ti_3_C_2_Tx is 2.7 times higher than that of mass-produced powdered activated carbon [100]. At the same time, the problem of resilience of MXene layers and an increase in the number of functional groups on them, as well as the problem of finding greener and more inexpensive methods for the synthesis of MXenes that exclude the use of toxic reagents, are barriers to the commercial use of MXenes for environmental purification [143].

Analysis of research papers demonstrates high potential for the use of MXenes in medical, biological and ecological applications, although any new materials can be employed in biomedicine only if their safety regarding human and animal health is undoubtedly proven. Unlike their bulk analogues, biocompatibility and biosafety of 2D nanomaterials, including MXenes, cannot be automatically derived from the properties of the comprising elements, as bioeffects of nanomaterials strongly depend on such characteristics as size, shape, dispersion, surface charge and hydrophilicity. Thus, one should have a very clear understanding of the interaction mechanisms between foreign nanostructured bodies and cells and tissues, and of possible adverse effects. Integrated safety assessment requires most thorough investigation of any possible interactions between nanomaterials and living systems. Special attention should be paid to all the possible effects of nanomaterial size, shape and various physico-chemical properties, as well as to various toxicity mechanisms. As MXenes are a recently created class of materials, discovered only in 2011, the information on their toxicity and biosafety is sporadic, while their biological activity still lacks comprehensive study. In the following sections we present an overview of a wide range of research papers on the toxicity of MXenes with different stoichiometry, namely, Ti_2_C and Ti_3_C_2_, studied both in vitro (bacteria, cell cultures) and in vivo (laboratory animals).

## 5. MXenes Toxicity In Vitro

Experimental data on MXene toxicity against human and animal cell cultures is extremely limited. Toxicity assessment of PEG-modified Ti_2_C MXene has been carried out against A375 human melanoma cells and MCF-7 human breast cancer cells, while HaCaT and MCF-10A were selected as nonmalignant cell lines. The cell cultures were incubated for 24 and 48 h with various doses of the studied MXene (0–500 mg/L) and were further analysed for potential morphological changes and cell membrane damage. The results show that HaCaT and MCF-10A retained about 70% viability at concentrations up to 500 μg/mL and preserved their normal status, which suggests acceptable biocompatibility. while A375 and MCF-7 cells displayed statistically significant increase in reactive oxygen species in them, that can be indicative of selective toxicity of the studied MXene towards cancerous cells [85]. Similar selective toxicity towards cancerous cells was observed in experiments with Ti_3_C_2_ using A549 and A375 cell culture [144].

Similar results indicating selective toxicity were obtained in Ti_2_NTx studies. The biocompatibility of Ti_2_NTx MXene was evaluated in vitro towards human skin malignant melanoma cells A375, human immortalized keratinocytes HaCaT, human breast cancer cells MCF-7, and normal human mammary epithelial cells MCF-10A. Ti_2_NTx was added to cell cultures at 62.5, 125, 250, 375 and 500 mg/L with subsequent 24 h incubation. Although Ti2NTx decreased viability of all the studied cell cultures, the studied MXene showed statistically higher toxicity towards cancerous cell lines in comparison to normal ones. The decrease in cell viabilities was dose-dependent and in some cases at higher Ti_2_NTx concentrations the toxicity towards cancerous cells was twice as high as towards normal cell lines [145].

Keeping in view cytotoxic MXene activity, Rozmysłowska-Wojciechowska et al. [146] carried out comparative toxicological assessment of pristine and collagen-modified Ti_3_C_2_ and Ti_2_C MXenes in order to develop MXenes with controlled cytotoxicity. The study was performed on human skin malignant melanoma cells A375, human immortalized keratinocytes HaCaT, human breast cancer cells MCF-7 and mammary epithelial cells MCF-10A, using the MTT assay. The cells were incubated for 24 h with 1, 5, 10, 25, 62.5 and 125 mg/L of MXenes. The obtained results demonstrated that higher MXene concentrations resulted in cell viability decrease in all the studied cultures. However, incubation in the presence of collagen-modified MXenes resulted in statistically significant increase in viability of all the cell cultures under study. Besides, the cytotoxic effect was more pronounced in malignant cells. The obtained results indicate that surface-modification with collagen reduces toxicity of MXenes in vitro.

Potential toxic effects can be reduced when the MXene surface is modified with soybean phospholipid, as demonstrated in the paper by Lin et al. using Ti_3_C_2_Tx MXene with soybean phospholipid (Ti_3_C_2_Tx-SP). Toxicity was assessed against mouse breast cancer cells 4T1 by means of a standard CCK-8 assay. The cells were incubated with various concentrations of Ti_3_C_2_-SP (400, 200, 100, 50, 25, 12, 6 and 0 μg/ml) for 24 and 48 h. The results show that Ti_3_C_2_-SP has a very insignificant effect on 4T1 cells viability, even at 400 μg/ml [63].

Yu et al. [147] synthesized Ti_3_C_2_ MXene quantum dots as a nanoagent for cancer photothermal therapy (PTT) applications. In vitro cytotoxicity was examined by means of an MTT assay on HeLa, MCF-7, U251 and HEK 293 cell cultures under 48h incubation in the presence of 6.25, 12.5, 25, 50 and 100 ppm of the studied MXene. The results showed no toxic effect in all the selected cell lines even at the highest MXene concentration of 100 ppm, which significantly exceeds the concentrations used in PTT applications.

Zong et al. [148] studied the effects of Ti_3_C_2_ MXene modified by the integration of GdW_10_-based polyoxometalates (GdW_10_@Ti_3_C_2_) during in vitro experiments. The study was performed on 4T1 mouse breast cancer cell culture. Incubation for 24 and 48 h with GdW_10_@Ti_3_C_2_ in various concentrations up to 500 ppm, revealed no toxic effect towards the cell culture, which indicates biocompatibility of the studied material.

A study with a multifunctional Ti_3_C_2_-based nanoplatform for doxorubicin, delivery and toxicity of Ti_3_C_2_ was examined against human colon cancer cell culture HCT-116. The material displayed no toxic effects, although the authors noted that their toxicity assessment might be not reliable as the bare Ti_3_C_2_ nanosheets heavily aggregate in physiological conditions [77].

Several papers [80,149] have indicated the absence of notable apoptosis or cytotoxic effect after cancer cells underwent treatment with Ti_3_C_2_ MXene with lateral dimensions of ~150–250 nm at concentrations in the range from 6 to 600 ppm.

Jastrzębska et al. [150] demonstrated that Ti_3_C_2_ MXene surface oxidation makes it possible to obtain selective cytotoxicity towards cancer cells as shown in experiments on MCF-7 human breast cancer cells and A375 human melanoma cells. The oxidized MXenes displayed no toxicity towards MCF-10A human mammary epithelial cells and HaCaT human keratinocyte cells used in the study for comparison.

Pan et al. [86] carried out the cytotoxicity assessment of the developed MXene-based matrix for tissue engineering that displayed zero toxic effect at MXene concentration up to 200 ppm, while low MXene concentrations (6 ppm) actively promoted cell proliferation.

The developers of Ti_3_C_2_ MXene-based hydrogel studied its in vitro toxicity against cultures of mouse hepatoma (HepAl-6), human hepatocellular carcinoma (SMMC-7721 and HepG2), human glioblastoma (U-118MG) and human astroglioma (U-251MG). The results indicated zero Ti_3_C_2_ impact on the cancer cells vitality [78].

Lin et al. [90] carried out toxicological studies of Nb2C MXenes nanosheets modified with polyvinyl pyrrolidone (Nb_2_C-PVP) against mouse mammary carcinoma 4T1 and human glioblastoma U87 cell lines. The material was added to the culture growth medium at 0, 12, 25, 50, 100 and 200 μg/ml, and was incubated for 24 and 48 h. The standard cell viability assay CCK-8 showed that Nb_2_C-PVP has a limited impact on the 4T1 and U87 cell viability even at concentrations of 200 μg/ml.

Thus, MXenes exhibit a wide range of in vitro biological effects from non-toxicity to total inhibition of cell growth. At the same time, MXenes are more toxic to bacteria, and safer for human cells. Researchers noted dose-dependent effects as well as changes in toxicity induced by other factors such as surface functionalization. Some studies indicate selective toxicity to cancer cells, although other studies do not confirm this.

## 6. MXene Toxicity In Vivo

Considering the large applicational potential of MXenes in biotechnology and biomedicine [151,152], risks of the materials for the environment and for living organisms must be thoroughly assessed.

Nasrallah et al. [153] carried out ecotoxicological assessment of Ti_3_C_2_Tx MXenes using a zebrafish embryo model. The acute toxicity of Ti_3_C_2_Tx was tested at concentrations of 25, 50, 100 and 200 μg/mL. According to the 96-hour sigmoidal mortality curve, the semilethal concentration LC_50_ of Ti_3_C_2_Tx was calculated to be 257.46 μg/mL. It was discovered that for Ti_3_C_2_Tx the lowest observed effect level (≥20% mortality) was 100 μg/mL, as this concentration caused a small increase in mortality (21%). However, no significant teratogenic effects were observed in the zebrafish embryos. Confirmation of this absence of toxicity was obtained through the locomotion and neurotoxicity assays, as Ti_3_C_2_Tx at 50 μg/mL had no adverse effects on neuromuscular activity. As the LC_50_ of Ti_3_C_2_Tx was greater than 100 μg/mL, it can be classified within the “practically nontoxic” group. Although one should note that the mortality rate in the group exposed to 100 μg/mL concentration was zero for 72 h and then suddenly spiked to 21%. This sudden increase in mortality can be attributed to Ti_3_C_2_Tx aggregation in tissues, which happens with time, eventually reaching the critical values for zebrafish embryos. It is also possible that aggregated MXenes can attach themselves to the embryo cell membranes and damage them. Large aggregates can cause sudden blockage to the minor capillary vessels, especially to the ones in the heart, thus causing death after a certain period of time. Thus, further examinations of Ti_3_C_2_Tx are required before its safety can be ascertained. Hussein et al. [154] discovered that Ti_3_C_2_Tx modification with Au particles strongly reduces the adverse effects displayed against zebrafish embryos (LC_50_ > 1000 µg/mL), down to complete absence of toxic or teratogenic manifestations.

Pan et al. [86] studied the influence of 3D composite scaffolds based on bioglass and Ti_3_C_2_ MXene on bone-tissue regeneration in Sprague–Dawley rats. The in vivo assessment of delayed toxicity was carried out after 24 weeks upon implantation. The results of hematological and histological examinations showed no significant alterations in the values compared to control, thus indicating absence of toxic effects from Ti_3_C_2_ MXene.

In order to study possible toxicity of Ti_3_C_2_ MXene-based quantum dots, in vivo research was performed on a Balb/c mice model by Yu et al. [147]. A single dose of the material was administered intravenously at 10 mg/kg. The results obtained from complete blood cell count and from histological examinations of heart, liver, spleen, lungs and kidneys, performed at 1, 7 and 14 days post-administration, showed zero toxic effects from the studied concentration. The authors are positive that these results should be attributed to their “green” synthesis method free from toxic organic solvents and components.

Han et al. [80] carried out an assessment of acute toxicity of Ti3C2-SP nanosheets upon intravenous administration of the material at 6.25, 12.5, 25, 50 mg/kg. Histocompatibility of the mice organs (heart, liver, spleen, lungs and kidneys) was evaluated upon days 1 and 7. No evidence of pathologies and significant histomorphological changes were observed in the examined organs compared to control, indicating no acute toxicity and adverse effects from Ti3C2-SP nanosheets administration. Excretion from the body rate and clearance routes was also studied. After 48 h excretion with urine and feces was 18.70% and 10.35%, respectively, indicating easy excretion of the studied material out of the body.

Dai et al. [149] studied in vivo biocompatibility and biosafety of MnOx/Ti_3_C_2_-SP composite after single-dose intravenous administration at 5, 10, and 20 mg/kg to healthy lab mice. As a result, upon the 30-day observation period all the major vital signs were normal, without deviation from the control. Further biochemical blood assay and the target organs examination revealed no signs of toxic action.

Zong et al. [148] studied the biosafety of Ti_3_C_2_ MXene nanosheets functionalized by the integration of GdW10-based polyoxometalates (GdW10@ Ti_3_C_2_) they themselves had developed, in an in vivo experiment on a female Kunming mice model. In order to evaluate excretion of the material out of the body, it was administered as a single dose at 5, 10 or 20 mg/kg, while the Ti content in urine and feces was measured 2, 6, 12, 24, 36 and 48 h after injection. According to the results, after 48 h Ti content in urine and feces was 9.1 and 38.2% of the injected amount, respectively. A subacute experiment was carried out on female Kunming mice in which the material was administered orally for a month. Examination of the overall condition, liver and kidney function tests, blood parameters, including mean corpuscular hemoglobin, mean corpuscular volume, hemoglobin, mean circulating platelet volume and white and red blood cell count revealed no significant toxic effects. Within the whole observation period, the mice displayed no visible alterations in their major vital signs or in their behavior. Histological examinations of the heart, liver, spleen, lungs and kidneys showed no pathological changes in the tissues.

In vivo studies on lab mice investigated toxicity of intratumoral injections of MXene-induced Ti_3_C_2_-containing hydrogels. The results showed that such gels are non-toxic and produce no negative effect on mice organs, including heart, liver, spleen, lungs and kidneys. The results of TNF-α, IL-6 and IL-1β assays did not deviate from the control values, which indicates zero immunotoxicity of the studied material [78].

Lin et al. [90] performed a toxicological assessment of polyvinyl pyrrolidone-modified Nb_2_C MXene nanosheets (Nb_2_C-PVP). The experiments were carried out on healthy Kunming mice. The animals were divided into four groups (n = 15): (1) control, (2) mice receiving Nb_2_C-PVP intravenously with subsequent NIR-I (808 nm) irradiation for 10 min, (3) mice receiving Nb_2_C-PVP intravenously with subsequent NIR-I (1064 nm) irradiation for 10 min, (4) mice receiving Nb_2_C-PVP intravenously under 24 h artificial daylight. In the three tested groups the dose was 20 mg/kg. Histological, hematological and biochemical blood parameters were examined 1, 7 and 24 days after injection. Hematological parameters of the animals from the experimental groups, including white and red blood cells count, platelet count, hemoglobin, mean circulating platelet volume and mean corpuscular hemoglobin stayed similar to those in the control throughout the experiment. Standard biochemical blood parameters, such as alanine transaminase (ALT), aspartate aminotransferase (AST), total protein, globulin, total bilirubin, blood urea nitrogen, creatinine (CREA) and albumen also remained within the control values. Thus, Nb_2_C-PVP in the studied dose produced no adverse effect on the blood chemistry values. Moreover, as ALT, AST and CREA are related functional parameters for kidneys and liver, one can assume that Nb_2_C-PVP has no significant nephro- and hepatotoxic action. Histological examinations of the heart, liver, spleen, lungs and kidneys showed no pathological changes in the tissues. The study of the excretion from the body rate and clearance routes revealed that 20% of Nb is excreted with urine and feces within 48 h. The results indicate high Nb_2_C-PVP biocompatibility.

Thus, most of the works indicate the absence of in vivo toxicity of MXenes. However, the results of long-term studies are not known yet. There are concerns that cumulative toxic effects may occur.

## 7. Toxicity Mechanisms

**Figure 9:**
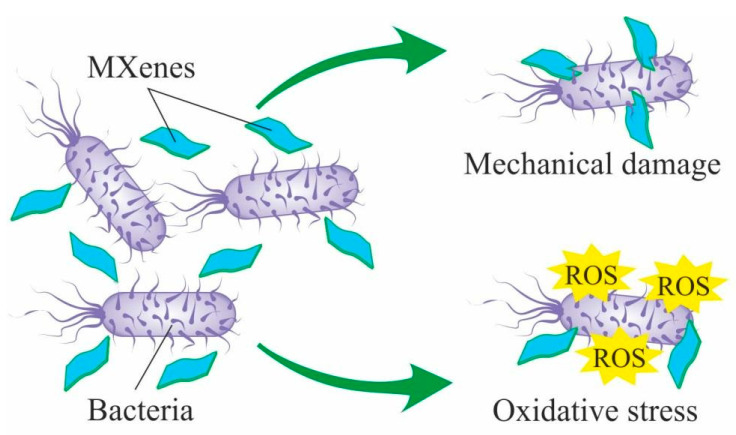
Experimentally confirmed mechanisms of MXenes cytotoxic effects on bacterial cells.

Among the major mechanisms responsible for MXenes toxicity against bacterial and animal cells observed in in vitro experiments, authors name oxidative stress [76,146,150] and mechanical damaging of the cell membrane with the sharp nanosheets edges [109,113] (Figure 9). These data are consistent with numerous studies of other insoluble 2D nanomaterials such as grapheme [155,156,157,158]. Given such data on genotoxicity and embryotoxicity of similar nanomaterials, the long-term effects of exposure to MXenes on living organisms should be studied very carefully. In studies conducted on bacterial cells, authors report that MXenes cytotoxic effects are more pronounced against Gram-positive than against Gram-negative bacteria, which might be attributed to cell membranes structural differences [109]. Additionally, most research on animal cells shows higher cytotoxicity towards malignant cells compared to normal cells. This effect may be connected with alteration in the subcellular internalization mechanism and with oxidative stress caused by active ROS generation [145].

Toxic effects have not been observed during acute and subacute in vivo experiments on lab mice and *Danio rerio* embryos. Nevertheless, it should be noted that the absence of these effects can be attributed to high aggregation rate of MXenes in biological fluids. Particle aggregation up to the critical size explains the time-dependent peak in the *Danio rerio* embryo mortality. It is also possible that aggregates can attach themselves to embryo cell membranes and damage them. Large aggregates can cause sudden blockage to the minor capillary vessels, especially the ones in the heart, thus causing death after a certain period of time [154].

Generally, there is no deep understanding of the mechanisms responsible for MXenes toxic effects both in vitro and in vivo, and the same is true for the majority of other nanomaterials. This lack of thorough knowledge can be attributed to the fact that nanotoxicity depends on a whole range of factors such as the particles shape and size, presence of defects and impurities [159], surface properties, and interaction with the biological environment, among others. Each of these characteristics contributes to general toxicity. At the same time, there exists great variability in the properties of formally similar nanomaterials fabricated either by different research teams, or by the same team but in the course of several synthesis cycles. The obtained materials are subsequently transferred into bioaccessible forms according to different approaches and protocols (e.g., the materials undergo dispersion in growth media or get turned into scaffoldings for adhesive cell cultures). Thus, not only identification of the precise toxicity factors and mechanisms, but even obtaining reproducible results often presents a challenge. Evidently, deep and comprehensive studies of this problem must be conducted in future.

Table 3 sums up the analyzed information on the biocompatibility/toxicity of MXenes both in vitro and in vivo.

## 8. Conclusions

Immediately after their discovery, MXenes became one of the most promising groups of 2D materials. Such properties as high specific surface area, high conductivity, absorption in the near-infrared region and their easy functionalization render them extremely promising for biomedical and environmental applications.

MXenes can become useful in bioimaging applications, including such methods as photoluminescence, photoacoustics, MRT and KT, as well as in tissue engineering, addressed drug delivery, and photo- and chemotherapy of cancer. They can also be employed as components of various sensors and sorbents, as well as antibacterial agents.

The results from most research works conducted on *E. coli*, *B. subtilis*, *S. aureus* and some others, indicate some antibacterial effects of MXenes. However, in other cases, the observed antibacterial action of these 2D materials is insignificant. This may be attributed to possible dependence of the MXenes toxicity against bacterial cells on the stoichiometry of the materials, as shown on the examples of Ti_2_C и Ti_3_C_2_.

The majority of papers studying MXenes effects on cell cultures report MXene biocompatibility, though with some exceptions. There are reports on significant MXene toxicity against A375 and MCF-7 human cancer cell cultures. Such selective toxicity may be attributed to the so-called enhanced permeability and retention effect. On the other hand, MXenes display no toxicity towards mouse mammary carcinoma 4t1 cells. It was noted that MXene modification with various substances can dramatically alter their biocompatibility/toxicity. There is data indicating no MXene toxicity in in vivo experiments on lab mice. The bulk of papers show that MXenes are excreted with urine and feces without noticeable accumulation in major organs. However, currently there are no research works assessing MXene safety via chronic experiments which could reveal the “weak spots” of their systemic penetration into human and animal organisms, as well as no studies using other mammals. At the same time, there are data on MXene material toxicity against Zebrafish embryos, which might indicate potential ecological hazard.

The difficulty in obtaining comparable results from different studies may be attributed, firstly, to the absence of standardized MXenes synthesis methods and techniques. The authors suggest essentially different and, as a rule, laboratory-scale synthesis approaches (top-down and bottom-up methods) using a variety of precursors with subsequent surface modification. Secondly, the fabricated MXenes differ in their physicochemical properties, including thickness of the obtained flakes, purity, number of surface defects, pH, and conductivity. Thirdly, the results received from different test objects are almost noncomparable. For example, in most cases, in vitro experiments on nonmalignant cells report some viability reduction, though to a minor degree [144,146,150], while the results of in vivo studies (at least, in acute and subacute experiments) display no toxic effects at the organismic or organ level [80,86]. This may be attributed to high 2D materials aggregation rate in biological fluids or to short exposure periods, insufficient for the development of more visible toxic effects. Myeloperoxidase-induced MXene biodegradation can also not be ruled out [90], or their biodegradation under the action of other enzymes, as well as activation of the systems protecting the organism against foreign substances.

Potential increase in the mass production of MXenes raises the question of their ecologicals, neither the toxicity rate, nor the life cycle of these materials in the environment and natural ecosystems have been studied. Powerful MXene-based adsorbents are being developed currently and prove highly effective for heavy metals removal from aquatic media [134,135,136,137,138,139,140,160]. However, uncontrolled release of MXenes into the environment will probably affect aquatic organisms [153] and bacteria. The situation is aggravated by constant addition of new types of Mxenes, as well as by new functionalizations of the current ones. Even the data obtained from in vitro and in vivo experiments provides no decisive answer whether MXenes are harmless or not. That is why it seems currently impossible to compare the results that various research groups have derived from studying MXenes formally belonging to the same type. Though Ti_3_C_2_Tx is considered to be the best studied MXene, the degree of understanding of its properties cannot be matched to that of graphene and other 2D materials. Judging from the data obtained for other 2D materials [156,161], one can conclude that on a long-term horizon MXenes are less safe than the results of acute experiments imply. We assume that the respiratory system, digestive tract and skin will be revealed as the systems most vulnerable to the adverse effects of MXenes, as is the case with other nanomaterials. In addition to exposure through medical instruments, drugs, and the environment, MXenes or their degradation products have the potential to affect humans through agricultural and food products, packaging materials, cosmetics, and a range of other consumer products. In addition, there is a risk of occupational diseases among workers in the emerging MXene industry.

In the future, the development of standardized test particles, as well as protocols for physicochemical characterization, nanoparticle (bio)interactions, and hazard assessment, is required to ensure convincing and reproducible results of the toxicological evaluation of MXenes, as well as other nanomaterials [162]. In addition to the classical methods for assessing toxicity in vitro and in vivo, it is advisable to use modern methods of nanotoxicology for the study of MXenes. This includes instrumental techniques such as precision-cut tissue slices, organ-on-chip, lateral flow immunoassay, high-throughput nanotoxicity screening, fluidic-based cell-on-chip, carbon fiber microelectrodes, biomimetic 3-d lung-on-a-chip, in vivo and ex vivo atomic force microscopy, among others. [163]. An emerging paradigm of prediction nanotoxicology also include the widespread use of computational methods based on including quantitative structure–activity relationship (QSAR) models, grouping/read-across approaches [164], molecular docking and molecular dynamics simulations [165]. These methods will enable the development of new applications of MXenes with rapid, inexpensive and bioethical studies of their safety.

**Figure 10:**
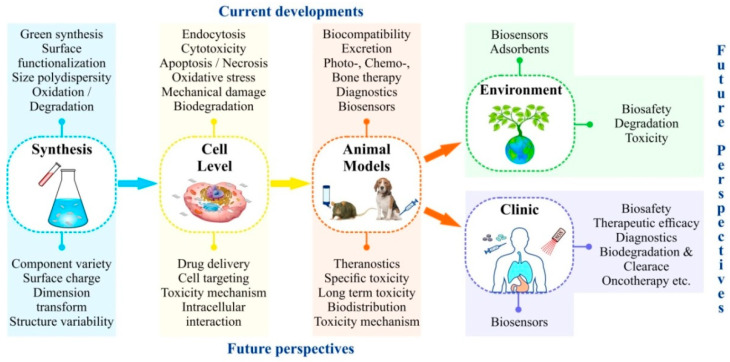
Current state and future prospects of MXene studies for biomedical and environmental applications.

Thus, MXenes could become breakthrough materials for numerous biomedical and environmental applications. Still, their employment is limited by the lack of experimental data. MXene-based systems for quick smart diagnostics are very promising. Development of tissue engineered scaffoldings that utilize mechanical strength, biocompatibility and electrical conductivity of MXenes might be an especially promising line of research. One expects emergence of novel MXene-based means for targeted drug and gene delivery, similar to those based on other 2D materials. Perhaps MXenes will be able to overcome all the shortcomings of other nanomaterials, but more detailed investigation of their toxicity mechanisms is required. Not only acute, but also, and perhaps more importantly, long-term influences, including mutagenic effects, produced by this type of nanomaterials on various biological organization levels, must be investigated. Deep studies should be carried out to disclose MXene bioaccumulation, biodegradation and behavior in the environment, taking into account their hydrophilic properties. Future studies presented in Figure 10 will certainly throw light on these issues.

## Figures and Tables

**Table 1:** MXenes based sensor for biomedicine.

Sensor Composition	Detectable Analyte	Sensor Type	Sensor Efficiency	Source
MXene-Ti_3_C_2_	Hemoglobin	Electrochemical biosensors	detection limit of 20 nM	[49]
MXene-Ti_3_C_2_	Hemoglobin	Electrochemical biosensor	linear range of 0.5–11,800 μM, detection limit of 0.12 μM	[57]
GOx/Au/Ti_3_C_2_T_x-_MXene/Nafion/GCE	Glucose	Electrochemical biosensor (amperometric)	detection limit of 5.9 μM	[50]
Ti_3_C_2_-MXene functionalized with aminosilane	Carcinoembryonic antigen (CEA)	Electrochemical biosensor	linear detection range of 0.0001–2000 ng mL^−1^ with sensitivity of 37.9 µA ng^−1^ mL cm^−2^ per decade	[51]
Ti_3_C_2_	Human papillomavirus (HPV)	Optical biosensor	detection limit of 100 pM	[58]
Ti_3_C_2_Tx MXene and phosphomolybdic acid embedded with polypyrrole	Osteopontin	Aptamer biosensor	0.98 μg/L	[60]
Ti_3_C_2_Tx/PtNP modified GCE	Ascorbic acid, dopamine, uric acid, acetaminophen	Electrochemical biosensor	nM level	[61]
OH-terminated Ti_3_C_2_	Label-free single-nucleotide in human urine	Electrochemiluminescence biosensor	Detection limit of 5 nM	[63]
Ti_3_C_2_Tx/Prussian blue	Glucose and lactate in sweat	Electrochemical biosensor (amperometric)	Sensitivities of 35.3 µA mm^−1^ cm^−2^ for glucose and 11.4 µA mm^−1^ cm^−2^ for lactate	[64]
MXene-Ti_3_C_2_Tx incorporated with a dialysis microfluidic chip	Urea, uric acid, and creatinine	Electrochemical biosensor		[66]
MXene-Ti_3_C_2_Tx modified screen-printed electrode	Acetaminophen (ACOP), isoniazid (INZ)	Electrochemical biosensor	Linear ranges from 0.25 to 2000 μM for ACOP and 0.1–4.6 mM for INZ. The detection limits of ACOP and INZ were 0.048 μM and 0.064 mM	[67]
Ti_3_C_2_Tx/ZIF-8	HIV-1 protein	Electrochemical biosensor	detection limit 0.3 fM	[68]
Au/Ti_3_C_2_T/HB5	HER2-positive cancer cells	Electrochemical cytosensor	linear range of 10^2^–10^6^ cells/mL, detection limit of 47 cells/mL	[69]
Chit/ChOx/Ti_3_C_2_Tx	Cholesterol	Electrochemical biosensor	concentration of cholesterol ranging from 0.3 to 4.5 nM, detection limit of 0.11 nM, sensitivity of 132.66 μA nM^−1^ cm^−2^	[70]
Ti_3_C_2_Tx MXene/LBG/PDMS	Cortisol	Electrochemical impedimetric immunosensor	linearity 0.01–100 nM, detection limit 88 pM	[71]
PEI-Ru@Ti_3_C_2_@AuNPs	SARS-CoV-2 RdRp gene	Electrochemiluminescent biosensor	Detection limit of 12.8 aM	[72]
ZnO/Ti_3_C_2_	Glucose	Electrochemical enzymatic biosensor	Sensitivity 29 μA mM^−1^ cm^−2^, limit of detection ≈ 17 μM, linear detection range 0.05–0.7 mM)	[73]

**Table 2:** MXenes-based sensor for environmental applications.

Sensor Composition	Detectable Analyte	Sensor Type	Sensor Efficiency	Source
Mo_2_Ti_2_AlC_3_/MWCNT	Bisphenol A	Electrochemical biosensor (amperometric)	0.01–8.50 μM	[128]
MXene-Ti_3_C_2_	Tyrosinase	Electrochemical biosensor	linear range from 0.05 to 15.5 μM L^−1^, detection limit of 12 nM L^−1^	[53]
MXene-Ti_3_C_2_	Ag^+^ and Mn^2+^	Optical sensors	Range of 0.1–40 μM for Ag^+^, detection limits of 9.7 nM; 0.5–60 μM for Mn^2+^ ions,	[59]
Ti_3_C_2_Tx (MXene)-modified glassy carbon electrode	BrO^3−^	Electrochemical biosensor	linear response from 50 nM to 5 μM, detection limit of 41 nM	[121]
Hydroxyl terminated alk-Ti_3_C_2_ modified GCE	Cd(II), Pb(II), Cu(II) and Hg(II)	Electrochemical biosensor	Detection limit of 0.098, 0.041, 0.032 and 0.130 μM for Cd(II), Pb(II), Cu(II) and Hg(II), respectively	[123]
AChE-Chit/Ti_3_C_2_-MXene/Au NPs/MnO_2_/Mn_3_O_4_/GCE	Organophosphorus pesticides	Electrochemical biosensor	concentration range (10^−12^–10^−6^ M), limit of detection (1.34 × 10^−13^ M)	[124]
AChE/CS-Ti_3_C_2_T_x_/GCE	Organophosphorous pesticides (malathion)	Electrochemical biosensor	concentration range of 10^−14^– 10^−8^ M, limit of detection 0.3 × 10^−14^ M	[125]
AChE/Ag@Ti_3_C_2_T_x_	Organophosphorous pesticides (malathion)	Electrochemical biosensor	concentration range of 10^−14^– 10^−8^ M	[126]
Ti_3_C_2_T_x_	carbamate pesticides (methiocarb and diethofencarb)	Electrochemical biosensor	detection limits were 0.19 μg mL^−1^ and 0.46 μg mL^−1^ for methiocarb and diethofencarb respectively	[129]
CdS/MXene-NH_2_/Zn_S_nO_3_	Cd^2+^, perfluorohexane	Photoelectrochemical biosensor	linear range of 0.008–100 nM, detection limit of 4.21 pM	[130]
MnMoO_4_–MXene-GCE	Hydroquinone, catechol	electrochemical biosensor	linear response from 5 nM to 65 nM, detection limit of 0.26 nM for Hydroquinone and 0.30 nM for catechol	[131]

**Table 3:** MXenes in vitro and in vivo toxicity.

MXene Composition, Concentration	Object under Study	Parameters under Study	Exposure Period	Effects	Source
In vitro					
Ti_2_C	*Bacillus* sp., *S. aureus, Sarcina*	colony growth inhibition (viability)	48 h	Non-toxic	[111,112]
Membranes containing single- and multilayer Ti_3_C_2_	*E. coli,* *B. subtilis*	Colony counting, viability	24 h	Growth inhibition by 73% in B. subtilis and by 67% in *E. coli*	[109,113]
Ti_3_C_2_Tx-chitosan, 0.75wt.% Ti_3_C_2_Tx	*E. coli и S. aureus*	Colony counting	4 h	Colony-forming units number reduction by 95% in *E. coli* and by 62% in *S. aureus*	[106]
Ti_3_C_2_Tx, Ti_3_C_2_Tx-Ag	*E. coli*	Colony counting (viability), cell morphology (SEM)	24 h	99% cell growth inhibition on the Ti_3_C_2_Tx-AgNP membrane and 60% on the non-functionalized Ti_3_C_2_Tx. Cell walls damage, disruptions	[116]
Ti_2_C-PEG, 0–500 mg/L	A375, MCF-7, HaCaT, MCF-10A	Cell viability (MTT assay), level of ROS, cell wall morphology (confocal laser scanning microscopy)	24, 48 h	Viability of HaCaT and MCF-10A >70%; A375, MCF-7 <20–40%. Nontoxic for HaCaT and MCF-10A, toxic for A375, MCF-7	[85]
Ti_3_C2, 0–500 mg/L	A549, MRC-5, A375, HaCaT	Cell viability (MTT assay, calcein-AM staining), level of ROS	24 h	Low toxicity for MRC-5, HaCaT, toxic for A549, A375	[144,145]
Ti_2_NTx, 0–500 mg/L	A375, HaCaT, MCF-7, MCF-10A	Cell viability (MTT assay), level of ROS, cells morphology (SEM)	24 h	Nontoxic for HaCaT and MCF-10A, toxic for A375, MCF-7	[145]
Ti_2_C, Ti_2_C + collagen, Ti_3_C_2_, Ti_3_C_2_ + collagen, 0–125 mg/L	A375, HaCaT, MCF-7, MCF-10A	Cell viability (MTT assay), level of ROS	24 h	Dose-dependent viability reduction in all the studied groups, Ti2C + collagen and Ti3C2 + collagen are less toxic than pure MXenes	[146]
Ti_3_C_2_Tx–SP, 0–400 mg/L	4T1	Cell counting (CCK-8 assay)	24, 48 h	Nontoxic	[87]
Ti_3_C_2_-QDs, 6.25–100 ppm	HeLa, MCF-7, U251, HEK 293	Cell viability (MTT assay)	48 h	Nontoxic	[147]
GdW10@ Ti_3_C_2_, 0–500 ppm	4T1	Cell counting (CCK assay)	24, 48 h	Nontoxic	[148]
Ti_3_C_2_- DOX, 0–100 mg/L	HCT-116	Cell viability (MTT assay)	24 h	Nontoxic	[77]
MnOx/Ti_3_C_2_ −SP, 0–100 mg/L	4T1	Cell counting (CCK-8 assay)	24, 48 h	Nontoxic	[149]
Ti_3_C_2_ −SP, 0–600 mg/L	4T1	Cell counting (CCK-8 assay)	12, 24, 48 h	Nontoxic	[80]
Ti_3_C_2_, 0–500 mg/L	A375, HaCaT, MCF-7, MCF-10A	Cell viability (MTT assay), level of ROS	24 h	Dose-dependent cell viability reduction. Low-toxic for HaCaT and MCF-10A, toxic for A375, MCF-7	[150]
Ti_3_C_2_-BG, 6–200 ppm	Saos-2, BMSCs	Cell counting (CCK-8 assay)		Nontoxic, under laser irradiation Saos-2 cell viability <40%. At 6 ppm stimulate BMSCs proliferation	[86]
Cellulose/Ti_3_C_2_ hydrogels, 0, 78.4, 156.8, 235.2, 313.4 ppm	HepAl-6, SMMC-7721, HepG2, U-118MG,U-251MG	CCK assay, calcein-AM staining	6, 24 h	Nontoxic	[78]
Nb2C-PVP, 0, 12, 25, 50, 100 и 200 μg/ml,	4T1, U87	CCK-8 assay	24, 48 h	Nontoxic	[90]
**In vivo**					
Ti_3_C_2_Tx, 25, 50, 100, 200 mg/L	Zebrafish embryo	Mortality, neurotoxicity	96 h	LC50-257, 46 mg/L	[153]
Ti_3_C_2_Tx-Au, 0, 50, 100, 200 mg/L	Zebrafish embryo	Mortality, body deformity, escoliosis, pigmentation, yolk edema, heart edema, movement defects	48, 72, 96 h	Nontoxic, LC50 > 1000 mg/L	[154]
Ti_3_C_2_-BG, 6–200 ppm	Rat Sprague–Dawley	Haematological, histological studies	Single dose	Nontoxic	[86]
Ti_3_C_2_ QDs, i.v. 10 mg/kg	Mice Balb/c	Haematological, histological studies	Single dose	Nontoxic	[147]
Ti_3_C_2_-SP, i.v. 6.25, 12.5, 25, 50 mg/kg	Mice Balb/c	Histological studies, metabolism, biodistribution	Single dose	Nontoxic; is excreted via urine and faeces	[80]
MnOx/Ti_3_C_2_-SP 5, 10, 20 mg/kg	Mice Kunming	Morphometric, haematological, histological studies	Single dose	Nontoxic	[149]
GdW10@ Ti_3_C_2_, orally 5, 10 or 20 mg/kg	Mice Kunming	Morphometric, haematological, histological studies	1 month	Nontoxic	[148]
Cellulose/Ti_3_C_2_ hydrogels	Mice BALB/c, C57BL/6	Morphometric, histological, haematological and immunology studies	Single dose	Nontoxic	[78]
Nb_2_C-PVP, 20 mg/kg	Mice Kunming	Histological, haematological and biochemical studies	Single dose	Nontoxic	[90]

## Data Availability

Not applicable.
